# Tendinous Inscriptions of the Rectus Abdominis: A Comprehensive Review

**DOI:** 10.7759/cureus.3100

**Published:** 2018-08-04

**Authors:** Rabjot Rai, Lilian C Azih, Joe Iwanaga, Marios Loukas, Martin Mortazavi, Rod J Oskouian, R. Shane Tubbs

**Affiliations:** 1 Anatomy, St. George's University School of Medicine, St. George's, GRD; 2 Hospital, Greater Los Angeles Hospital, Los Angeles, USA; 3 Medical Education and Simulation, Seattle Science Foundation, Seattle, USA; 4 Anatomical Sciences, St. George's University, St. George's, GRD; 5 Neurosurgery, National Skull Base Center, Thousand Oaks, USA; 6 Neurosurgery, Swedish Neuroscience Institute, Seattle, USA; 7 Neurosurgery, Seattle Science Foundation, Seattle, USA

**Keywords:** anterior abdominal wall, segmentation, rectus abdominis muscle, anatomy, tendon

## Abstract

The rectus abdominis muscles are interrupted by tendinous inscriptions, which typically appear as fibrous bands crossing the muscle. The current literature on tendinous inscriptions is scarce; hence, this review will provide a detailed overview of their anatomical description, including their variation, embryology, comparative anatomy, and clinical application. Understanding the anatomy and function of the tendinous inscription assists in providing clinical relevance and in guiding reconstructive surgeons in their surgical planning and design.

## Introduction and background

Despite numerous reports on the rectus abdominis (RA) muscle, there has been scarce literature describing the tendinous inscriptions, also known as tendinous intersections or inscriptions tendinae of the RA. Surgeons encounter this area frequently during anterolateral abdominal wall procedures, including transverse RA myocutaneous (TRAM) flap reconstruction, and as such, a thorough knowledge of the regional anatomy and the variations in tendinous inscriptions is vital for surgical planning.

## Review

Detailed review of tendinous inscriptions and anatomical variations

The RA encompasses a fibrous sheath extending from the entirety of the anterior abdominal wall to the superior two-thirds of the muscle. The RA originates from the pubic symphysis and inserts into the fifth to seventh costal cartilages and the xiphoid process.

**Figure 1:**
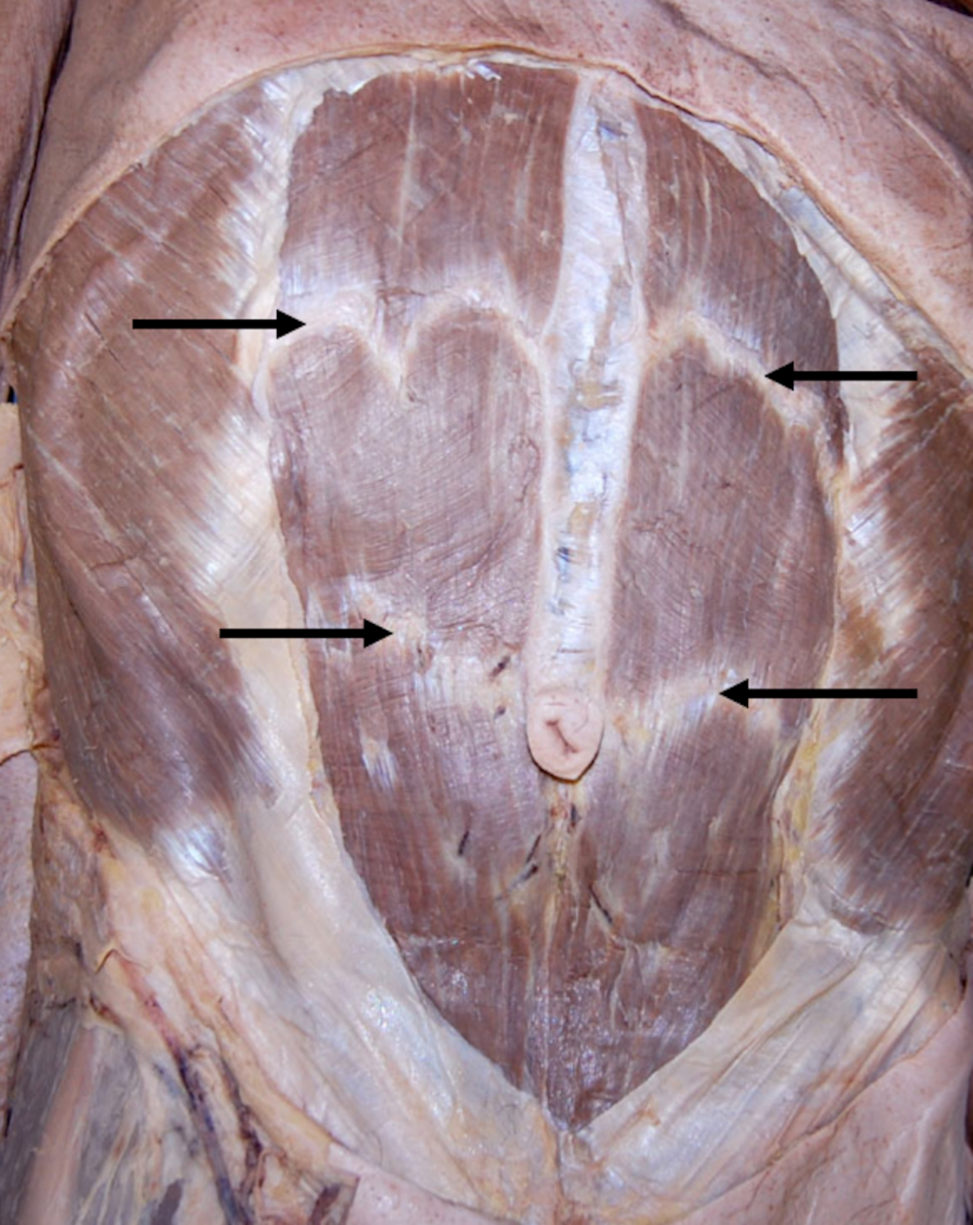
Anterior abdominal wall of a cadaver noting the tendinous intersections (arrows). Note the difference in shape between the upper left and right intersection.

**Figure 2:**
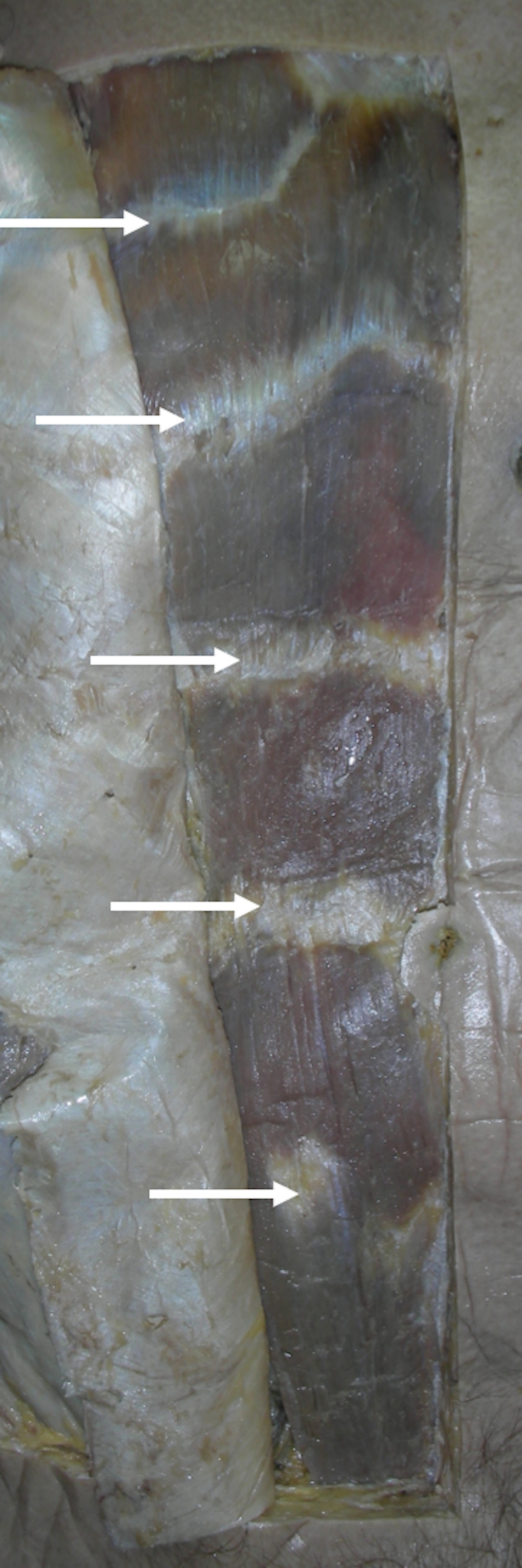
Anterior abdominal wall of a cadaver noting five intersections on the dissected right side. A gastrostomy tube is noted as reference.

The muscle facilitates multiple movements, including the flexion and extension of the truck, respiratory mechanics, and maintaining posture [1]. The RA is divided, in part, by tendinous inscriptions that are usually three to five irregular fibrous bands (Figures 1-2) that cross the RA and adhere mainly to the anterior surface of the sheath [2]. In anatomical studies, the tendinous inscriptions are usually paired and demonstrated to course in the oblique or transverse plane [3]. The tendinous inscriptions limit fluid collection beneath the anterior rectus sheath, prevent muscle rupture, and aid in the biomechanics of the RA [1-2].

The tendinous inscriptions may divide the RA into three or four muscular segments: at the opposite free end of the xiphoid process, umbilicus, midway between these two structures, and sporadically below the umbilicus [1,3]. Although typically the RA has three to four paired tendinous inscriptions, there are multiple variations in their arrangement. Anson and McVay [2] examined 162 RA muscles in 81 cadaveric specimens and found that all specimens ranged from having at least one tendinous inscription to none having more than four inscriptions. They concluded the following anatomical variations in the tendinous inscriptions: 1.2% (2/162) of the muscles had one inscription, 5.9% (9/162) two inscriptions, 58.0% (94/162) three inscriptions, 35.2% (57/162) four inscriptions, and 82% of the specimens had an equal number of inscriptions bilaterally [2]. Other studies showed similar results, with the majority of RA muscles having three paired tendinous inscriptions [1,3-4].

Grossly, the tendinous inscriptions appear to span the entire width of the RA, but in reality, these vary in length. Anson and McVay [2] found that out of the 530 inscriptions studied, only 67% (356/530) completely extended without interruption from the lateral to the medial muscle borders, 24% (128/530) did not completely extend across more than half the width of the muscle, and 9% (46/530) were fragments that made up less than half the width of the muscle with the majority of these fragments touching the lateral muscle border [2]. Broyles et al. [1] examined the thickness of the tendinous inscriptions extending from the anterior to the posterior layer of the rectus sheath. Out of 32 fresh human cadavers, a total of 168 tendinous inscriptions were assessed. An analysis of the 168 tendinous intersections illustrated that 18% (30/168) of these inscriptions showed full thickness during their anterior–posterior course without intervening the neurovascular structures. Out of the 32 fresh human cadavers, nine had an absent or damaged hemiabdominal anatomy, and thus, only the intact contralateral abdomen was analyzed; of these nine hemiabdominal specimens, 55 tendinous inscriptions were assessed. Broyles et al. [1] concluded that 42% (23/55) of the cadaveric hemiabdomens demonstrated at least one full-thickness tendinous inscription. This is of clinical importance as the epigastric arteries and veins course posteriorly and through the full-thickness inscriptions and hence require consideration during abdominal dissection and flap elevation [1].

Tendinous inscriptions can also be classified based on their unique shapes and characteristics. Milloy et al. [5] examined 484 complete or almost complete tendinous inscriptions and classified them into four types: 109 (22.5%) were grouped as (A) based on their straight lines; 57 (12%) were grouped as (B)–those which were biphasic with their apices pointing caudally; 186 (38%) were grouped as (C)–those which were biphasic with their apices pointing cephalad; and 132 (27.5%) grouped as (D)–the multiphasic type [1,3,5]. A thorough understanding of their anatomy and variations is important to surgeons who operate this region [6-11].

Microscopic anatomy

In an attempt to learn about the composition of the tendinous inscriptions and their association with the RA and the anterior abdominal wall, Lange [12] found that a muscle disc, which encases the RA from cranial to caudal without interruption at its dorsal side, also covered every inscription. When examining the fibers that form the inscriptions directly, Lange [12] found that the fibers within this disc lay in a parallel fashion, with some of the fibers deviating and running in a twisted pattern and changing direction from ventral to dorsal and then dorsal to ventral. He also established that the diameter of the dorsal muscle fibers increased from cranial to caudal. In his further examination of the dorsal sides, the muscle fibers were covered by the posterior part of the RA, which in of itself is only lightly connected by soft connective tissues. Lange [12] reported that the actual connection between the dorsal muscle fibers and the inscriptions was provided by the soft connective tissues, which start at the dorsal end of the muscle and extend to the ventral side, are arranged like chambers, and fold into the inscriptions on the ventral part of the muscle. Lange [12] established that the inscriptions are built from cranial to caudal tendons of the muscular in-between segments that are interconnected together. Of note, the connection within the anterior layer of the rectus sheath and the muscle was strong enough to prevent separation by blunt dissection alone [12]. He also described the functional relationship between the fatty connective tissues that connect the tendinous inscriptions to the RA as the tendinous inscriptions aid in gliding movements. This was observed as the contractions of the lateral abdominal muscles can be transferred via the anterior leaf of the rectus sheath and the tendinous inscriptions, thus offering structural stability during the contraction and varying degrees of forward flexion to the lumbar region for the vertebral column [12].

Vascular supply

The vascular anatomy of the tendinous inscriptions is vital during surgical procedures, such as TRAM flaps; gaining knowledge of the vascular architecture allows for the maintenance of improved circulation. Whetzel and Huang [4] proposed a study to explain the vascular anatomy of the tendinous inscriptions of the RA by dissecting 14 cadavers. Their results concluded that the arterial supply arises from a system of transverse arcades surfacing from the superior or inferior epigastric arteries. It was also noted that the superficial and deep arcades coursed parallel within the tendinous inscriptions. The deep arcades provided muscular branches superiorly and inferiorly, while the superficial arcades branched off perforators toward the overlying skin alongside muscular branches. Whetzel and Huang [4] also mapped the perforators of the RA and tendinous inscriptions and concluded that the greatest number of perforators was found within the tendinous inscriptions. The periumbilical inferior tendinous inscription region contains the highest density (1.47 per sq cm), while the superior periumbilical muscular segment contains the highest concentration (0.123 per sq cm) [4].

Embryology

The RA is divided by tendinous inscriptions into serially arranged bilateral compartments, which are only present on the anterior surface of the RA and fuse with the anterior layer of the rectus sheath. Leading up to the 17th week of gestation, the RA muscles of human fetuses lack tendinous inscriptions. Between the late 17th and 20th weeks of gestation, the tendinous inscriptions begin to appear and this variation is theorized to be due to mechanical factors [13]. The embryological origins of tendinous inscriptions are uncertain; however, it is suggested that these inscriptions represent the myosepta outlining the myotomes that fused to form the muscles [1,3]. Thus, the division of the RA muscle by inscriptions increases its strength [1,3,14]. This speculation is further reinforced by Brown and McGill’s examination of the anterior abdominal wall connective tissues [15], including the internal abdominal oblique aponeurosis and tendinous inscriptions, which found that the tendinous inscriptions function to provide strength to the RA by acting as anchor points along its length. As such, the absence of the tendinous inscriptions as reported in a case by Ikiz et al. [13] may contribute to resisting abdominal wall herniation, as, consequentially, the absence of these inscriptions may weaken the anterior abdominal wall.

Microscopic samples of inscriptions from both embryos and adults were examined by Pongs (1937), who was able to demonstrate an ontogenetic restructuring and concluded that the main premise for the wedge is to form the ventral surface of the rectus [12]. 

In a comparative anatomical embryological study conducted by Rizk and Adieb [16], the development of the anterior abdominal wall in 60 rat embryos and 10 postnatal rat specimens was examined. The authors concluded that the tendinous intersections of the rectus muscle were only observed postnatally and attached exclusively to the ventral—not the dorsal—rectus sheath. Rizk and Adieb concluded that due to the lack of prenatal findings of the tendinous inscriptions, the embryological origins of the tendinous inscriptions represent the intermediate tendons of a multigastric longitudinal muscle column instead of being remnants of the original segmentation of myotomes [16].

Comparative anatomy

The presence of tendinous inscriptions in the RA of various species demonstrate differences in their structural anatomy. A study conducted by Manzano et al. reported amphibians, such as *Rana pipiens*, support tendinous inscriptions in order to connect the RA to the skin via the connective tissues. However, amphibians, such as *Phyllomdusa boliviana*, make use of the RA directly—not by inscriptions alone—to adjoin the internal surface of the skin [17]. Another comparative anatomy of the tendinous inscriptions of the RA muscle was reported in a study by Lancaster and Henson [18], noting its presence in the bat species, *Pteronotus parnelli*. They reported that the rectus sheath adheres to the tendinous inscriptions at the caudal border of the pectoral muscles. 

Scapino [19] discussed digastric muscles in carnivores, noting that numerous authors attempted to determine the presence of tendinous inscriptions in carnivores, and if present, whether they completely divided the abdominis muscle into two. He reported conflicting results regarding the presence, partial presence, or absence of tendinous inscriptions in various species, including canids, ursids, procyonids, mustelids, viverrids, delids, felids, pinnipeds, and among some genera and classes of these groups. The tendinous inscriptions found in the digastric muscles in carnivores were identified as being thin; Scapino attributes this to assist in maximizing muscle fiber length—which speed and large excursions are contingent upon. The fine nature of the tendinous inscription during gross dissection made visualization of the structure difficult. Scapino also hypothesized the lack of visualization of the tendinous inscription due to it being anatomically concealed by the muscular bellies that adhere to them [19].

Clinical applications

Autologous breast reconstruction commonly involves using myocutaneous pedicle flaps, which include the TRAM flap and latissimus dorsi flap [20]. The harvesting of these flaps is in part due to the vascular architecture, allowing for an optimal vascularity and hence a successful flap. As mentioned, the vascular supply of the tendinous inscription is characterized by transverse arcades arising from either the superior or inferior epigastric arteries, which give off branches to supply the muscle and the overlying skin. A greater number of perforators per square centimeter originate from the tendinous inscriptions compared to the rest of the RA [4]. Boyd et al. [21] demonstrated that the highest concentration of perforators was in the paraumbilical area; however, Whetzel and Huang [4] determined that the highest density of perforators was found in the tendinous inscriptions, particularly the periumbilical inferior tendinous inscription [1,4,21]. Thus, the perforators in the tendinous inscriptions are significant for the blood supply of the anterior abdominal wall. Hence, keeping this in mind, the surgical planning and design of the TRAM flaps must take into consideration both the location of the paraumbilical area and the vascular supply to the tendinous inscriptions for the best surgical outcomes [4].

**Figure 3:**
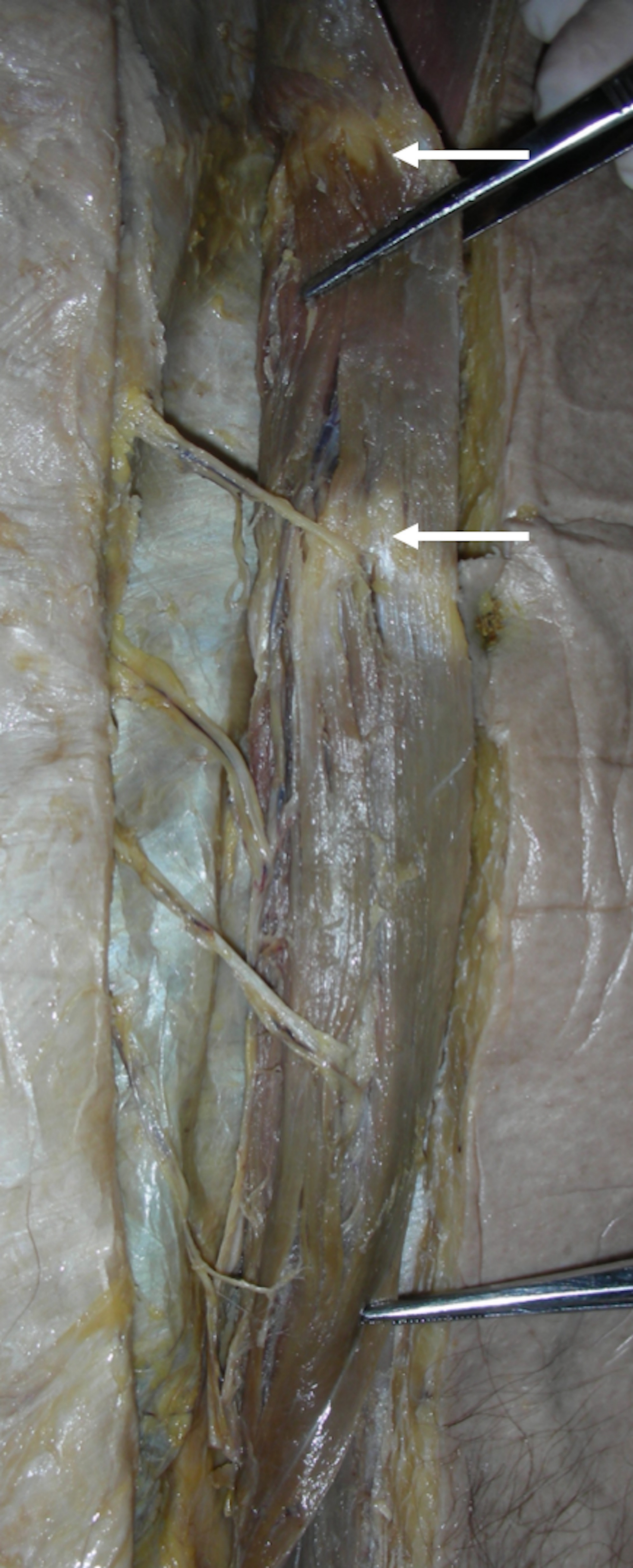
Reflection of the right-sided rectus abdominis demonstrates the neurovascular pedicles. A gastrostomy tube is noted as reference.

**Figure 4:**
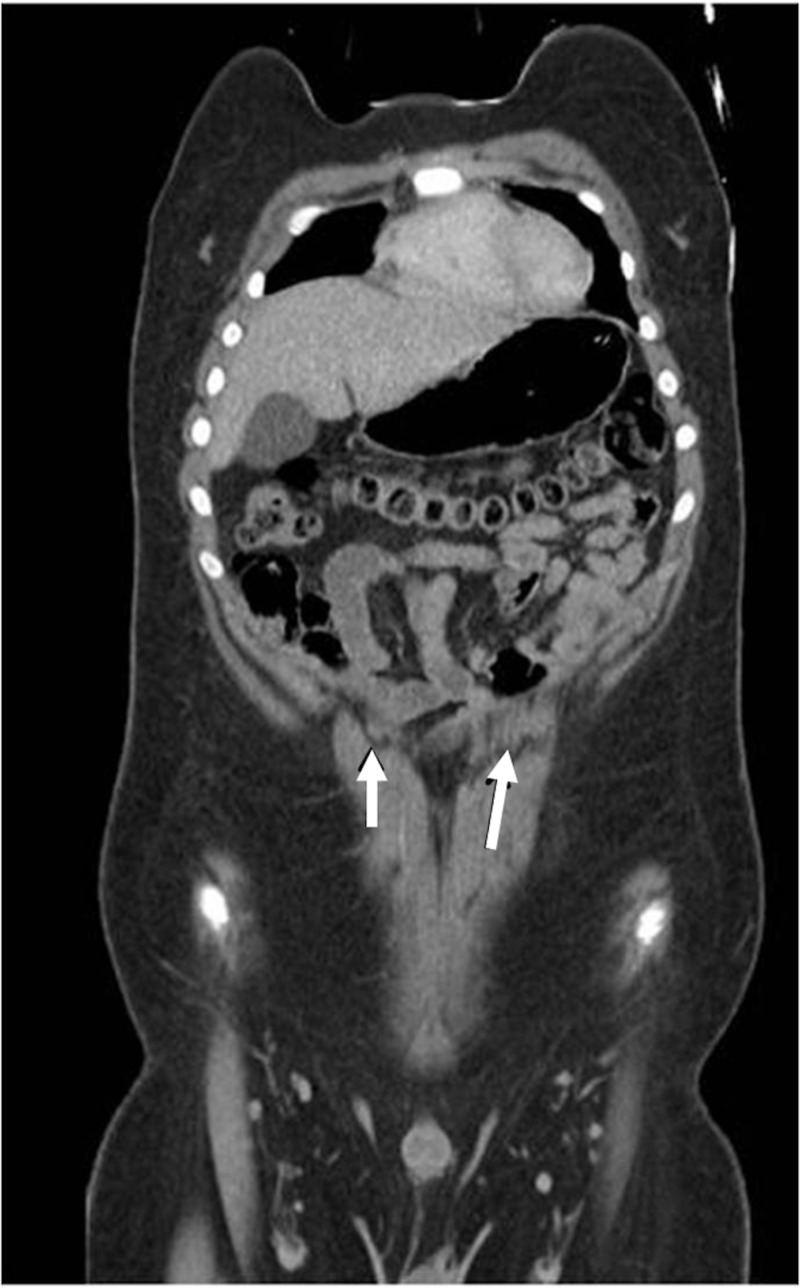
MRI of the anterior abdominal wall. Note the tendinous intersections (arrows). MRI: magnetic resonance imaging

The unusual patterns of tendinous inscriptions of the RA have a distinct surgical importance to the reconstructive surgeon. Das et al. [22] studied 46 human cadavers over a three-year period and their studies resulted in the discovery of two unusual patterns of the tendinous inscriptions—one with tendinous inscriptions arched and the other with the inscriptions at different levels on the right and left sides of the same cadaver. Based on their studies, Das et al. found a functional relationship between the tendinous inscriptions and the RA, such that the arched pattern discovered in their studies functioned to provide better biomechanics to the RA muscle instead of the normal transverse pattern. Interestingly, Das et al. also found that the tendinous inscriptions are the primary sites of anastomosis of numerous blood vessels (Figure 3), a finding that is of use to the breast reconstructive surgeon whose awareness of these unusual patterns should aid in design of the TRAM flaps. These same principles can be applied in other reconstructive surgeries, such as with component separation when used in the repair of large ventral hernia defects of the anterior abdominal wall. These inscriptions can also be visualized on imaging, such as magnetic resonance imaging (MRI; Figure 4) [22].

The reconstruction of the breast and the extremities with the RA myocutaneous free flaps has been widely used. The versatile soft-tissue nature and the presence of long, large-diameter inferior epigastric arteries and veins allow for ideal microvascular anastomoses when working with the RA free flaps. This resourceful free flap can also be used in head and neck reconstruction procedures. For instance, in oral cavity reconstruction, such as during total glossectomy, the goal of surgical reconstruction is to bring sufficient bulk to the neotongue. This will improve palate approximation and aid in articulation and swallowing. These RA flaps are used when the mandibular arch via bony reconstruction is not needed. The flaps allow for successful reconstruction mainly due to two reasons: the rectus sheath and tendinous inscription. These structures allow the defect to be affixed laterally and assist in suspending the soft tissues within the oral cavity [23]. 

Inscriptions seen in other areas of the body

There are three classifications for the intramuscular arrangements of skeletal muscles: (1) the muscle fibers extend the entire length of the fascicle from insertion; (2) muscle fibers with tendinous inscriptions link muscle fibers from end to end; and (3) intricate organization of long parallel-fibered muscles in an overlapping array where fibers may course the fascicle partially and cease intrafascicularly [24]. As such, other areas of the human body can have tendinous inscriptions such as the semitendinosus muscle, semispinalis capitis, splenius capitis, and the internal/external abdominal obliques muscles [24-27]. 

The semitendinosus muscle is one of the three hamstring muscles located in the posterior thigh and is divided by tendinous inscriptions into two parts [28]. A study conducted by Kellis et al. [25] demonstrated the effect of the tendinous inscriptions on the function of the semitendinosus muscle. Kellis et al. conducted an in vivo and in vitro examination of the tendinous inscriptions of the human semitendinosus muscle in 18 young males and found that all measured angles of the tendinous inscriptions of the semitendinosus muscles, with the exception of the angle between the tendinous inscription arm and the deep aponeurosis, increased significantly from rest to maximum voluntary contraction. This indicated that the role of the tendinous inscription lies in its connection with the muscle fascicles, such that the semitendinosus muscle contracts as one unit during contraction. Kellis et al. reported that the morphology of the tendinous inscriptions changes during rest to maximum voluntary contraction, thereby allowing a non-uniform displacement to occur and aid in movement. Therefore, an association between the fascicles and tendinous inscription assists in the normal functioning of the semitendinosus muscle [25].

The semispinalis capitis muscle originates from the articular processes of C4 to C6 and the transverse processes of T1 to T6, at times T7, and inserts into the occipital bone between the inferior and superior nuchal lines. It mainly has tendinous inscriptions dividing the muscle at the level of the C6 vertebra. Infrequently, another tendinous inscription may be found at the level of the C2 vertebra marked in the medial fibers arising from the thoracic vertebrae. These inscriptions of the semispinalis capitis divide the muscle into three endplate zones with one in every third of the muscle length. Due to the differing fiber lengths in the lower third of the semispinalis capitis muscle, the endplate zone is more widely distributed, which aids the semispinalis capitis muscle functional activities in extension and antigravity control of the head when one leans forward [26]. There are also three large dorsal muscles in the neck crossed with tendinous inscriptions: the splenius, biventer cervices, and complexus. The inscriptions intersect the muscles as connective tissue bands and are a functional component of the physiology of the neck muscles. The neck muscles come in long and short fibers, in which the short fibers insert between tendinous inscriptions and the long fibers attach to tendinous inscriptions and perforate them. Besides acting as insertion points, the tendinous inscriptions house numerous Golgi tendon organs. The inscriptions are perforated or lie adjacent to many of the spindles of the neck muscles [24].

The internal abdominal oblique muscle lies between the external abdominal oblique and the transversus abdominis [27]. The lumbar triangle (triangle of Petit) is an area that, although variable in size, is between the posterior margin of the external abdominal oblique, the lateral margin of the latissimus dorsi, and the crest of the ilium. In this area, the internal abdominal oblique is subcutaneous; however, variations in the attachments and the extent of development of the fleshy part of the muscle may be significant. Occasionally, the tendinous inscriptions are found within the muscle, perhaps indicating a primitive segmental condition [27].

## Conclusions

Our collective review is based on the findings of various studies and aims to present a more detailed anatomical review of the tendinous inscriptions of the RA, including its variations, embryological origin, and comparative anatomy. Understanding the anatomy and function of the tendinous inscriptions aids in providing clinical relevance and in guiding reconstructive surgeons in their surgical planning and design.
